# Identification of cell surface markers for acute myeloid leukemia prognosis based on multi-model analysis

**DOI:** 10.7555/JBR.38.20240065

**Published:** 2024-05-29

**Authors:** Jiaqi Tang, Lin Luo, Bakwatanisa Bosco, Ning Li, Bin Huang, Rongrong Wu, Zihan Lin, Ming Hong, Wenjie Liu, Lingxiang Wu, Wei Wu, Mengyan Zhu, Quanzhong Liu, Peng Xia, Miao Yu, Diru Yao, Sali Lv, Ruohan Zhang, Wentao Liu, Qianghu Wang, Kening Li

**Affiliations:** 1 Department of Bioinformatics, School of Biomedical Engineering and Informatics, Nanjing Medical University, Nanjing, Jiangsu 211166, China; 2 Department of Hematology of the Affiliated Huai'an No. 1 People's Hospital of Nanjing Medical University, Northern Jiangsu Institute of Clinical Medicine, Huai'an, Jiangsu 223300, China; 3 Collaborative Innovation Center for Personalized Cancer Medicine, Jiangsu Key Lab of Cancer Biomarkers, Prevention and Treatment, Nanjing Medical University, Nanjing, Jiangsu 211166, China; 4 Department of Hematology, the First Affiliated Hospital of Nanjing Medical University, Jiangsu Province Hospital, Nanjing, Jiangsu 210029, China; 5 Key Laboratory of Hematology of Nanjing Medical University, Nanjing, Jiangsu 210029, China; 6 Department of Pharmacology, School of Basic Medical Sciences, Nanjing Medical University, Nanjing, Jiangsu 211166, China; 7 The Affiliated Cancer Hospital of Nanjing Medical University, Jiangsu Cancer Hospital, Jiangsu Institute of Cancer Research, Nanjing, Jiangsu 210002, China

**Keywords:** acute myeloid leukemia, cell surface markers, prognosis, drug sensitivity, multi-model analysis

## Abstract

Given the extremely high inter-patient heterogeneity of acute myeloid leukemia (AML), the identification of biomarkers for prognostic assessment and therapeutic guidance is critical. Cell surface markers (CSMs) have been shown to play an important role in AML leukemogenesis and progression. In the current study, we evaluated the prognostic potential of all human CSMs in 130 AML patients from The Cancer Genome Atlas (TCGA) based on differential gene expression analysis and univariable Cox proportional hazards regression analysis. By using multi-model analysis, including Adaptive LASSO regression, LASSO regression, and Elastic Net, we constructed a 9-CSMs prognostic model for risk stratification of the AML patients. The predictive value of the 9-CSMs risk score was further validated at the transcriptome and proteome levels. Multivariable Cox regression analysis showed that the risk score was an independent prognostic factor for the AML patients. The AML patients with high 9-CSMs risk scores had a shorter overall and event-free survival time than those with low scores. Notably, single-cell RNA-sequencing analysis indicated that patients with high 9-CSMs risk scores exhibited chemotherapy resistance. Furthermore, PI3K inhibitors were identified as potential treatments for these high-risk patients. In conclusion, we constructed a 9-CSMs prognostic model that served as an independent prognostic factor for the survival of AML patients and held the potential for guiding drug therapy.

## Introduction

Acute myeloid leukemia (AML) is a malignant clonal disorder characterized by myeloid blast proliferation with expansion and a block in differentiation ^[ 1– 2] ^. Chemotherapy has been the predominant treatment for AML for decades. Although most patients initially respond to chemotherapy, approximately 75% of the patients relapse and die from the disease within five years of diagnosis ^[ 3] ^. Additionally, the molecular heterogeneity among AML patients may lead to varying treatment outcomes ^[ 4– 6] ^. Therefore, it is essential to identify molecular markers at the time of diagnosis to predict the risk of treatment failure or relapse, as well as survival outcomes of AML patients ^[ 7] ^.

Several clinical features are shown to be associated with AML prognosis, including age, white blood cell count, and a history of hematological diseases ^[ 8] ^. On the other hand, certain biological and cytogenetic characteristics of leukemia cells, such as internal tandem repeats of the *FLT3* gene, as well as t(8;21), inv(16), and 3q abnormalities, may also help predict prognosis ^[ 9] ^. Although these factors have been applied to patient prognostic stratification, some patients still do not have identifiable European LeukemiaNet (ELN) 2022 guideline-defined mutations or cytogenetic events. As such, the prognostic value of these mutations or cytogenetic characteristics is limited ^[ 10] ^. Therefore, the risk stratification and prognosis evaluation of AML patients need to be further improved.

Cell surface markers (CSMs) are proteins present on the surfaces of cells, serving as crucial identifiers that distinguish and categorize various cell types. Because of their extracellular accessibility and involvement in regulating cellular processes, such as metabolite transport and intercellular communication, CSMs have been widely used as diagnostic and prognostic markers in cancers ^[ 11] ^. For example, CSMs have been used to identify cancer stem cells, whose persistence after chemotherapy causes cancer relapse, and for immunophenotyping in cancers ^[ 12] ^. Numerous studies suggest that CSMs play a crucial role in the prognostic assessment and prediction of disease progression. Moreover, it has been reported that over 66% of drugs approved for treating human diseases target CSMs, based on the data from the DrugBank database ^[ 13] ^. The identification of cancer-specific CSMs may not only suggest disease progression and stratify patients into different prognostic categories, but also provide targets for drug therapy.

In AML, CSMs have been reported to be applicable for screening, diagnosis, prognosis, predicting treatment response, and monitoring disease progression. Notably, several studies showed that CSMs, such as CD34, CD38, CD96, and CD47, might have prognostic significance to further guide treatment decisions ^[ 14] ^. However, these studies primarily examined the prognostic significance of individual CSM in AML and frequently relied on a small size of patient samples ^[ 15]^ and low throughput technologies such as flow cytometry ^[ 16] ^, immunohistochemistry, and quantitative polymerase chain reaction ^[ 17] ^. Because of the complexity of AML and its extremely high inter-sample heterogeneity, the expression level of a single CSM may have a limited classification power for risk stratification. Therefore, a comprehensive computational study evaluating the prognostic potential of human CSMs in AML patients may provide more precise information for risk stratification and prognostic predictions for AML patients.

To comprehensively evaluate the prognostic efficiency of all CSMs in AML patients, we conducted multi-model analyses on high-throughput transcriptome data using the LASSO, Adaptive LASSO, and Elastic Net algorithms. Consequently, we developed a 9-CSM prognostic model with potential applications in risk stratification and prognostic prediction for AML patients. Furthermore, this model was validated at the protein level, to provide a novel perspective on prognostic prediction and therapeutic decision-making for AML patients.

## Materials and methods

### Data acquisition and processing

The information on CSMs was downloaded from the Cell Surface Protein Atlas (CSPA, http://wlab.ethz.ch/cspa/), a free online database that describes 2886 proteins constituting the human in silico surfaceome.

The clinical and microarray data (GSE147515 contains data from both 198 normal subjects and 1534 AML patients, while GSE10358, GSE12417, GSE71014, GSE84334, GSE106291, GSE193094, GSE146173, and GSE183817 contain data from 91, 74, 104, 38, 235, 6, 233, and 7 AML patients, respectively) were downloaded from the Gene Expression Omnibus (GEO, http://www.ncbi.nlm.nih.gov/geo/). The gene expression profiles and their corresponding clinical information of the BeatAML were downloaded from the Beat acute myeloid leukemia database (BeatAML, http://www.vizome.org/aml/), which includes 239 AML patients. The proteomics data related to BeatAML were downloaded from the Genomic Data Commons Data Portal (GDC, https://pdc.cancer.gov/pdc/). The proteomics data related to The Cancer Genome Atlas (TCGA)-Acute Myeloid Leukemia-like (LAML) were downloaded from the deep-scale proteomic and phosphoproteomic database of AML ( https://proteomics.leylab.org/). The clinical information and the gene expression profiles of the TCGA-LAML were downloaded from TCGA ( https://portal.gdc.cancer.gov/). We used the average gene expression level for multiple probes corresponding to one single gene. The information on 130 patients for the TCGA training data and the GEO external validation datasets is shown in ***Supplementary Table 1*** (available online).

### The expression and function analyses of CSMs in AML

We used the limma package to identify genes that were differentially expressed between AML and normal samples, calculated the mean absolute deviation, and performed principal component analysis (PCA) using the R in-built function "prcomp" to obtain the matrix of principal components. The "enrichr" function implemented in the "enrichR" package was used to identify significantly enriched biological pathways and processes.

### Training and construction of the 9-CSMs prognostic model

The Elastic Net, LASSO, and Adaptive LASSO regression methods were implemented using the "glmnet" R package (version 4.1.3) to select candidate features. The LASSO proposed by Tibshirani *et al* is a technique used to filter out genes with a significant association with patient survival ^[ 18] ^. The LASSO transforms based on the formula below,

1 $ \hat{\beta }^{ l a s s o} = {}_{\beta}^{arg min }\left(\frac{1}{2 n}\|y - X \beta\|_2^2+\lambda {\sum}_{j=1}^p\left|\beta_j\right|\right) .$

Then, separately, we subjected the CSMs to the Adaptive LASSO regression, which was proposed by Zou *et al* ^[ 19] ^. The Adaptive LASSO applies different weights for different parameters, as shown below,

2 $\hat{\beta}^{ {adaplasso }}={}_{\beta}^{arg min }\left(\frac{1}{2 n}\left\|y- X \beta\right\|_2^2+\lambda {\sum}_{j=1}^p \omega_j\left|\beta_j\right|\right).$

The Elastic Net regression method proposed by Hastie combines the benefits of $\lambda_1 $ and $\lambda_2 $ regularizations ^[ 20] ^, as shown below,

3 \begin{equation*}\begin{split} 
 \hat{\beta }^{ { elasticNet }}= {}_{\beta}^{arg min }\left(\frac{1}{2 n}\left\|y- X \beta\right\|_2^{2 }+\lambda_1 \|\beta\|_1+\lambda_2\|\beta\|_2^2\right).

\end{split}\end{equation*}

The union of the candidate features selected from each method was then subjected to an automatic model averaging and multi-model selection analysis implemented using the "glmulti" function. The best model was selected based on the minor Akaike information criterion (AIC) value. Finally, the prognostic risk score was calculated using the following equation:

4 $ score=\sum_{i=1}^N\beta_i\times Gene_i, $

where *β _i_* is the CSM model coefficient obtained from the best model ( ***Supplementary Table 2 ***, available online) and *Gene _i_* is the gene expression level of each CSM in the prognostic model.

We performed a Kaplan-Meier survival analysis of the overall survival (OS) and event-free survival (EFS) of AML patients using the "Survival" and "Survminer" R packages. For each dataset, the patients were classified into high-risk and low-risk groups according to the median CSMs score, and the significance was evaluated by the log-rank test. To evaluate the predictive performance of our prognostic model, we performed receiver operating characteristic (ROC) curve analysis using the "timeROC" R package (version 0.4) for 1-year, 3-year, and 5-year survival. The area under the curve (AUC) was used to estimate the accuracy of the model.

### Evaluation of the independence of the 9-CSMs prognostic model

We performed univariable Cox regression analysis using the "coxph" function to evaluate the prognostic score of the 9-CSMs model. Furthermore, we performed multivariable Cox regression analysis on variables, including the 9-CSMs prognostic score, age, sex, race, cytogenetic risk, molecular subtype, bone marrow blast percentage, PB blast percentage, WBC count, and gene mutations, to examine their potential association with the survival outcome of AML patients in the TCGA cohort.

### Construction and calibration of the nomogram model

For the nomogram model, we integrated independent prognostic factors obtained from multivariable Cox regression analysis together with survival time data and event data to fit the Cox proportional hazards model using the "survival" package. The nomogram model for predicting the OS rate of patients with AML was constructed by "foreign" and "rms" R packages. Finally, calibration plots were generated for respective survival times, with the x-axis showing predicted survival and the y-axis showing actual OS.

### Gene-gene interaction network analysis

To unravel the gene-gene interactions associated with 9-CSMs, we used the GeneMANIA Database ( http://genemania.org/), a comprehensive database that contains information about known and predicted protein-protein interactions, to find genes that may share functions.

### Pre-processing and analysis of single-cell RNA sequencing (scRNA-seq) data

The scRNA-seq data was downloaded from the OMIX ( https://ngdc.cncb.ac.cn/omix, accession No. OMIX002180). Data integration, unsupervised clustering, and visualization were conducted using the Seurat framework ^[ 21] ^. We identified the cellular states of the leukemia-like cells according to the article by Li *et al* ^[ 22] ^. The risk score was computed through AUCell.

### Drug sensitivity analysis

To evaluate the use of our prognostic model in guiding medication for patients with AML, we examined the association between anticancer drug sensitivity and the prognostic model risk score. We obtained the half maximal inhibitory concentration (IC _50_) data and the cell line expression data from the Genomics of Drug Sensitivity in Cancer database (GDSC, https://www.cancerrxgene.org/). The Spearman's rank correlation test was performed to compute the correlation coefficient and *P*-values between the IC _50_ values and the prognostic model risk score. A Spearman's rank adjusted *P*-value < 0.05 was considered statistically significant.

### Prediction of immunotherapy response

We used the online tool Tumor Immune Dysfunction and Exclusion (TIDE, http://tide.dfci.harvard.edu/) to predict the response of AML patients to the existing immune checkpoint inhibitors (ICIs) targeting PD-1 and CTLA-4 ^[ 23– 24] ^. We inputted the gene expression matrix of AML patients, and downloaded the corresponding analysis results from the website. The Spearman's rank correlation test was used to compute the correlation coefficient and *P*-values between the TIDE score and the 9-CSMs risk score.

## Results

### Construction of the 9-CSMs prognostic model based on multi-model analysis

**Figure 1:**
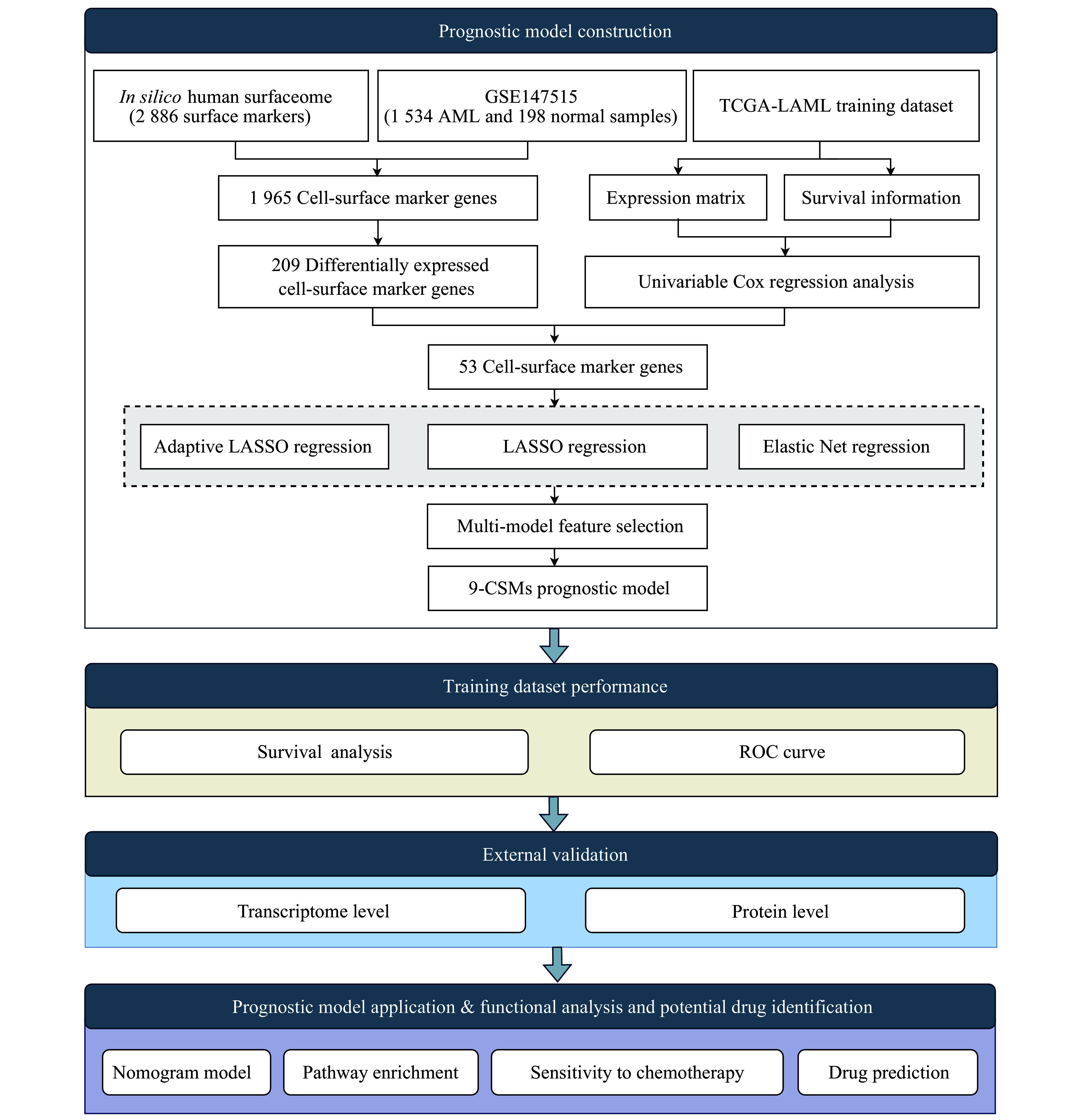
Schematic of the workflow for identifying and constructing the CSM prognostic model.

**Figure 2:**
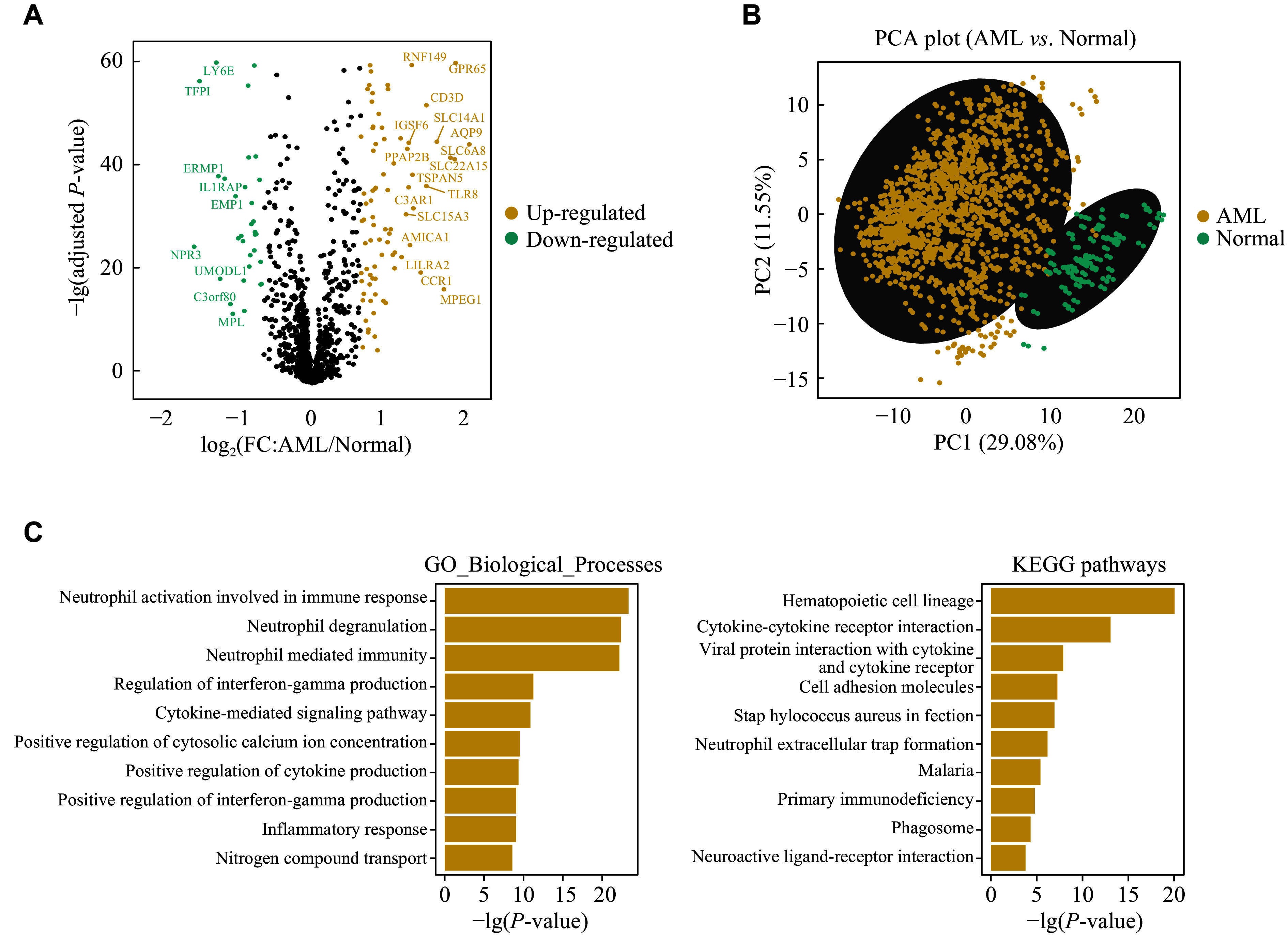
Differential gene expression and functional enrichment analysis between AML and normal samples.

To identify reliable predictive markers for the prognosis of AML patients, we designed a bulk RNA-seq-based workflow to comprehensively dissect the characteristics of AML patients ( ***Fig. 1***). We obtained 2886 CSMs from the CSPA database and then identified 209 CSMs that were differentially expressed in AML ( *n* = 1534) and healthy control ( *n* = 198) samples ( ***Fig. 2A***, ***Supplementary Tables 3*** and ***4*** [available online]). The results of PCA showed that the differentially expressed genes mentioned above effectively distinguished AML from normal tissues ( ***Fig. 2B***). Further functional enrichment analysis revealed that CSMs up-regulated in AML were enriched in biological processes associated with the inflammatory response, cytokine-cytokine receptor interaction, IL-6/JAK/STAT3 signaling pathway, and IL-2/STAT5 signaling ( ***Fig. 2C***), indicating that the up-regulated cell-surface proteins in AML may be associated with the immune response and tumor development.

**Figure 3:**
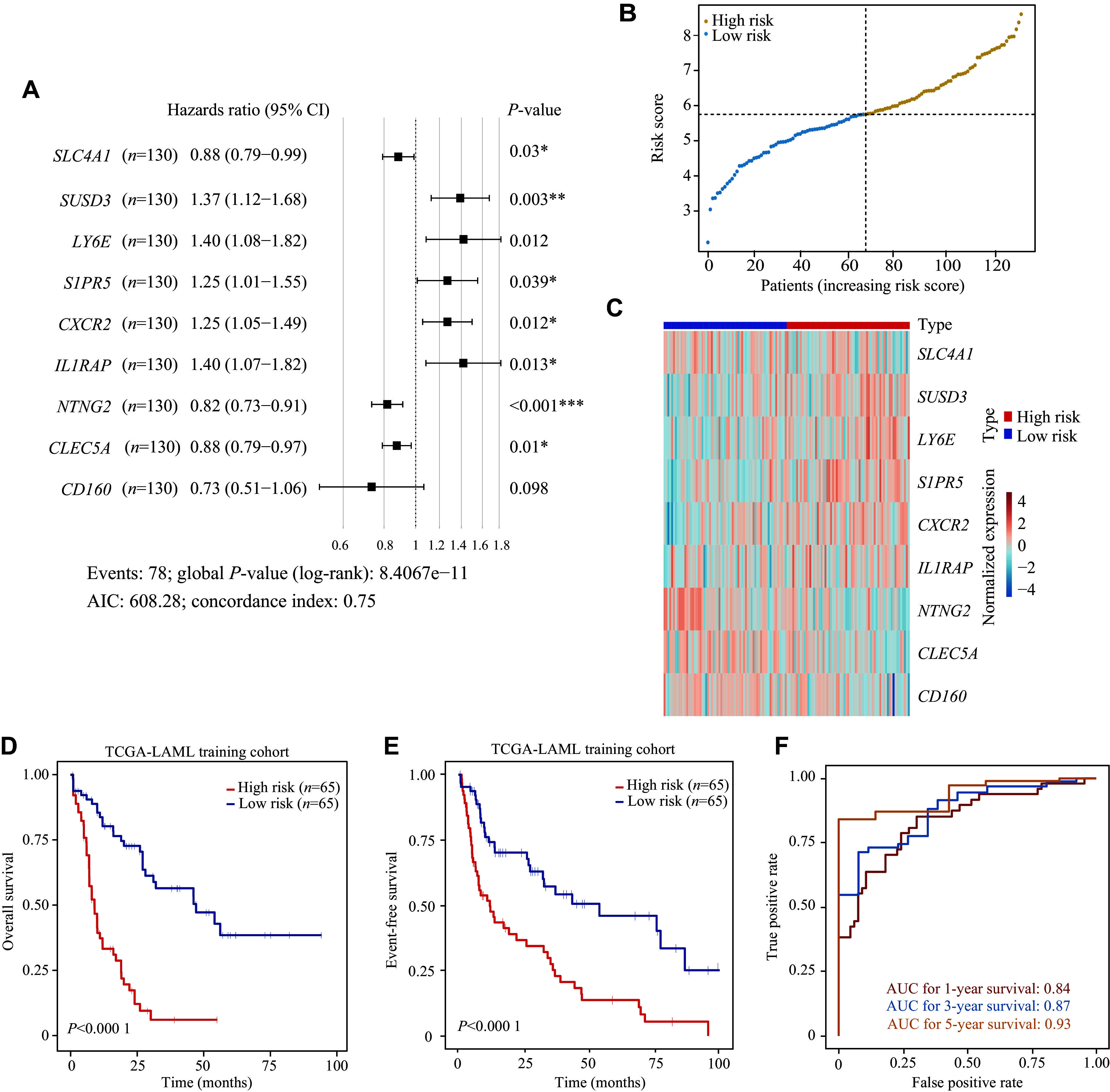
Construction and evaluation of the 9-CSMs prognostic models in the training datasets.

The univariable Cox regression analysis was performed to evaluate the association between gene expression levels of these differentially expressed CSMs and OS of 130 AML patients in the TCGA-LAML cohort ( ***Supplementary Table 5 ***, available online). We then performed multi-model feature selection analysis using three regression analysis methods: LASSO regression analysis, Adaptive LASSO, and Elastic Net. We implemented this analysis using a 10-fold cross-validation maximum likelihood penalty estimator in the TCGA-LAML training cohort. The optimal λ values selected by each method are shown in ***Supplementary Fig. 1*** (available online). In addition, CSMs selected by the three methods were combined to perform an automated model average and multi-model selection analysis based on the smallest AIC value ( ***Supplementary Tables 6 ***– ***9 ***, available online). Using the multivariable Cox regression, the best model (9-CSMs) was determined ( ***Supplementary Table 2 ***, available online). The results of the stepwise multivariable Cox regression analysis showed that the identified markers, except for *CD160*, were independent prognostic factors for AML patient survival ( ***Fig. 3A***). The details of the 9-CSMs model are shown in ***Supplementary Table 10*** (available online). Furthermore, *CXCR2,* *LY6E*, *SUSD3*, *S1PR5*, and *IL1RAP* were identified as risk factors, while *CD160*, *CLEC5A*, *NTNG2*, and *SLC4A1* were identified as protective factors in the prognostic model.

### The 9-CSMs prognostic model predicted outcomes in the training dataset

To assess the prognostic performance of the model, we assigned a risk score calculated from the 9-CSMs prognostic model to each patient in the TCGA-LAML training cohort ( ***Supplementary Table 11 ***, available online) and classified these patients into the low-risk and high-risk groups based on the median 9-CSMs score ( ***Fig. 3B***). In patients with high-risk scores, each risk factor was highly expressed, while each protective factor was highly expressed in patients with low-risk scores ( ***Fig. 3C***). The 9-CSMs prognostic model significantly predicted survival outcomes for both OS and event-free survival of 130 AML patients in the Kaplan-Meier survival analysis ( ***Fig. 3D*** and ***3E***), demonstrating that patients with high-risk scores had shorter survival times than those with low risk scores in the TCGA-LAML training cohort.

Additionally, we assessed the predictive performance of the 9-CSMs prognostic model by calculating the area under the time-dependent ROC in the TCGA-LAML training cohort. The AUC for OS at 1, 3, and 5 years was 0.84, 0.87, and 0.93, respectively, indicating that the model for the TCGA-LAML training cohort performed well ( ***Fig. 3F***).

### Validation of the 9-CSMs prognostic model at the transcriptome and proteome levels

**Figure 4:**
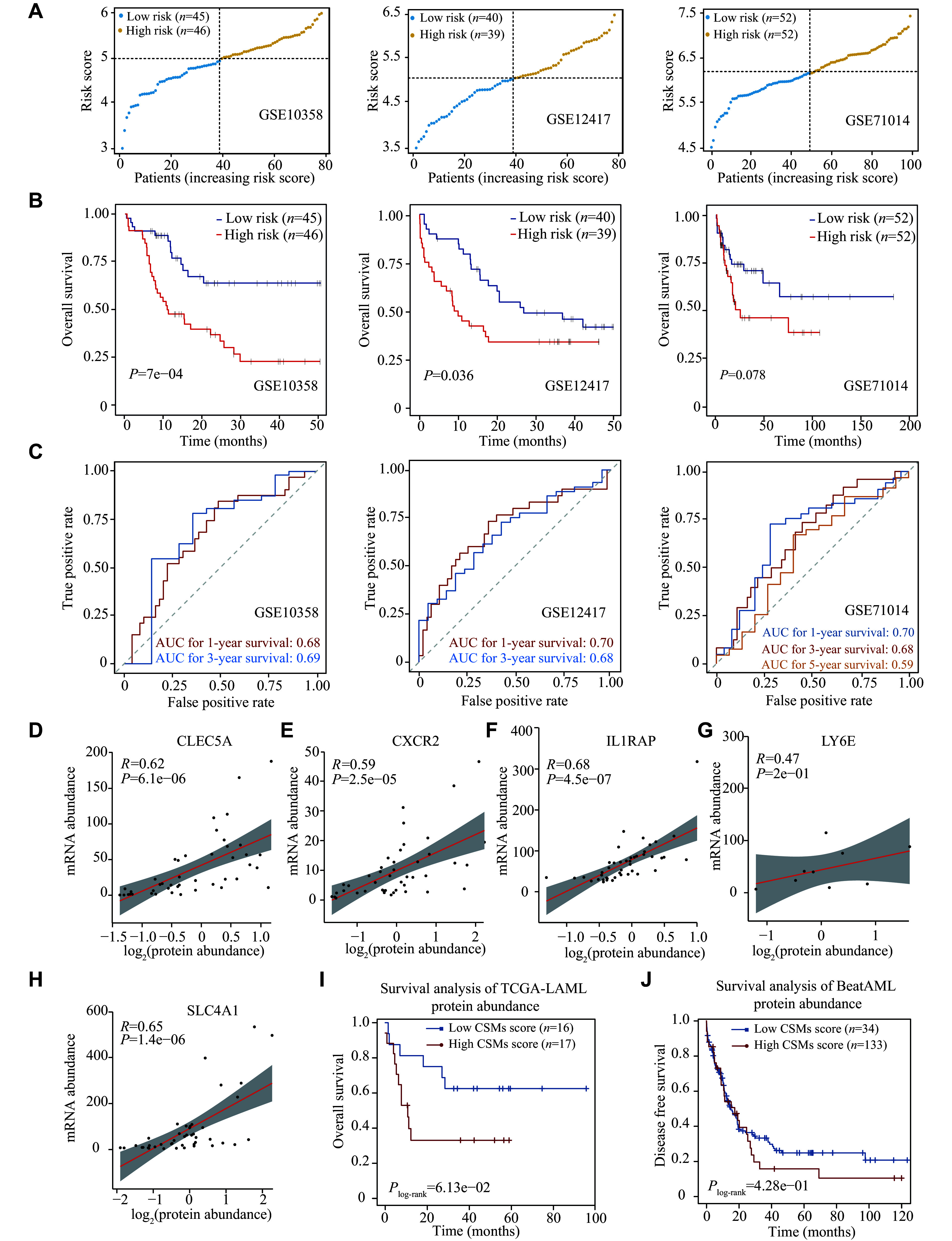
Evaluation of the 9-CSMs prognostic model in multiple validation cohorts and its prognostic value at the protein level.

We validated the predictive performance of the 9-CSMs prognostic model in three independent validation datasets, including GSE10358, GSE12417, and GSE71014 ( ***Supplementary Tables 12 ***– ***14 ***, available online). Patients were classified into the low-risk and high-risk groups according to the median 9-CSMs scores ( ***Fig. 4A***). The Kaplan-Meier survival analysis consistently revealed that patients had significantly poorer survival outcomes in the high-risk group than in the low-risk group ( *P* = 0.0007 for GSE10358, *P* = 0.036 for GSE12417, and *P* = 0.078 for GSE71014, ***Fig. 4B***), indicating that patients with high-risk scores had shorter survival than those with low-risk scores in all three datasets. We then performed time-dependent ROC analysis to further assess the consistency of the predictive ability of the 9-CSMs prognostic model in the three independent datasets and found that the 9-CSMs prognostic model accurately predicted the survival outcomes of AML patients ( ***Fig. 4C***).

Only five genes, including *CLEC5A*, *CXCR2*, *IL1RAP*, *LY6E*, and *SLC4A1*, had available proteomic data in the TCGA-LAML dataset, and the results showed a significant correlation between their mRNA and protein abundances ( ***Fig. 4D***– ***4H***). Consequently, we calculated the risk score using the five available proteins in the TCGA-LAML dataset (33 AML patients) and found that the CSMs risk score effectively stratified patient outcomes at the protein level ( ***Fig. 4I***). We also found a trend towards poorer prognosis in patients with higher 9-CSMs risk scores in another independent cohort, BeatAML, which includes 167 AML patients ( ***Fig. 4J***).

### The 9-CSMs risk score was an independent prognostic factor for AML patients

To evaluate whether the 9-CSMs prognostic model was an independent prognostic factor for patient survival, we performed univariable and multivariable Cox regression analyses on clinicopathological variables, along with the 9-CSMs risk score in the TCGA-LAML training cohort. The results showed that the 9-CSMs risk score, *TP53*, *DNMT3A*, *RUNX1*, and age were independent prognostic factors for AML patients ( ***Table 1***). These independent prognostic factors ( *i.e.*, age, *TP53*, *DNMT3A*, *RUNX1*, and 9-CSMs risk score) were further used to construct a nomogram to facilitate AML patient prognosis prediction. The corresponding score for each factor was calculated in the nomogram and the total score might be used as a tool for survival prediction for AML patients ( ***Supplementary Fig. 2A ***, available online). The prediction results of the nomogram calibration curve of OS were consistent with the observation results of AML patients ( ***Supplementary Fig. 2B ***).

**Table 1:** Univariable and multivariable Cox regression analyses for independent prognostic factors in 130 TCGA-LAML patients

Characteristics	*n*	Univariable Cox regression analysis			Multivariable Cox regression analysis	
		Hazards ratio (95% CI)	*P-*value		Hazards ratio（95% CI)	*P*-value
Age (years)						
<60	75	Ref				
≥60	55	2.74 (1.72–4.36)	<0.0010		2.27 (1.40–3.68)	<0.0010
Cytogenetic risk						
Favorable	30	Ref				
Intermediate	72	3.19 (1.56–6.50)	0.0010		0.93 (0.55–1.59)	0.7991
Poor	26	3.79 (1.67–8.61)	0.0010			
TP53						
Wild type	120	Ref				
Mutant	8	0.20 (0.09–0.43)	<0.0010		0.26 (0.11–0.63)	0.0030
DNMT3A						
Wild type	97	Ref				
Mutant	31	0.42 (0.25–0.70)	0.0010		0.45 (0.26–0.77)	0.0040
RUNX1						
Wild type	115	Ref				
Mutant	13	0.5 (0.26–0.95)	0.0350		0.45 (0.23–0.91)	0.0257
9-CSMs risk score		1.73 (1.43–2.10)	<0.0010		1.75 (1.05–2.92)	0.0324
Abbreviation: CI, confidence interval; CSMs, cell surface markers.						

**Figure 5:**
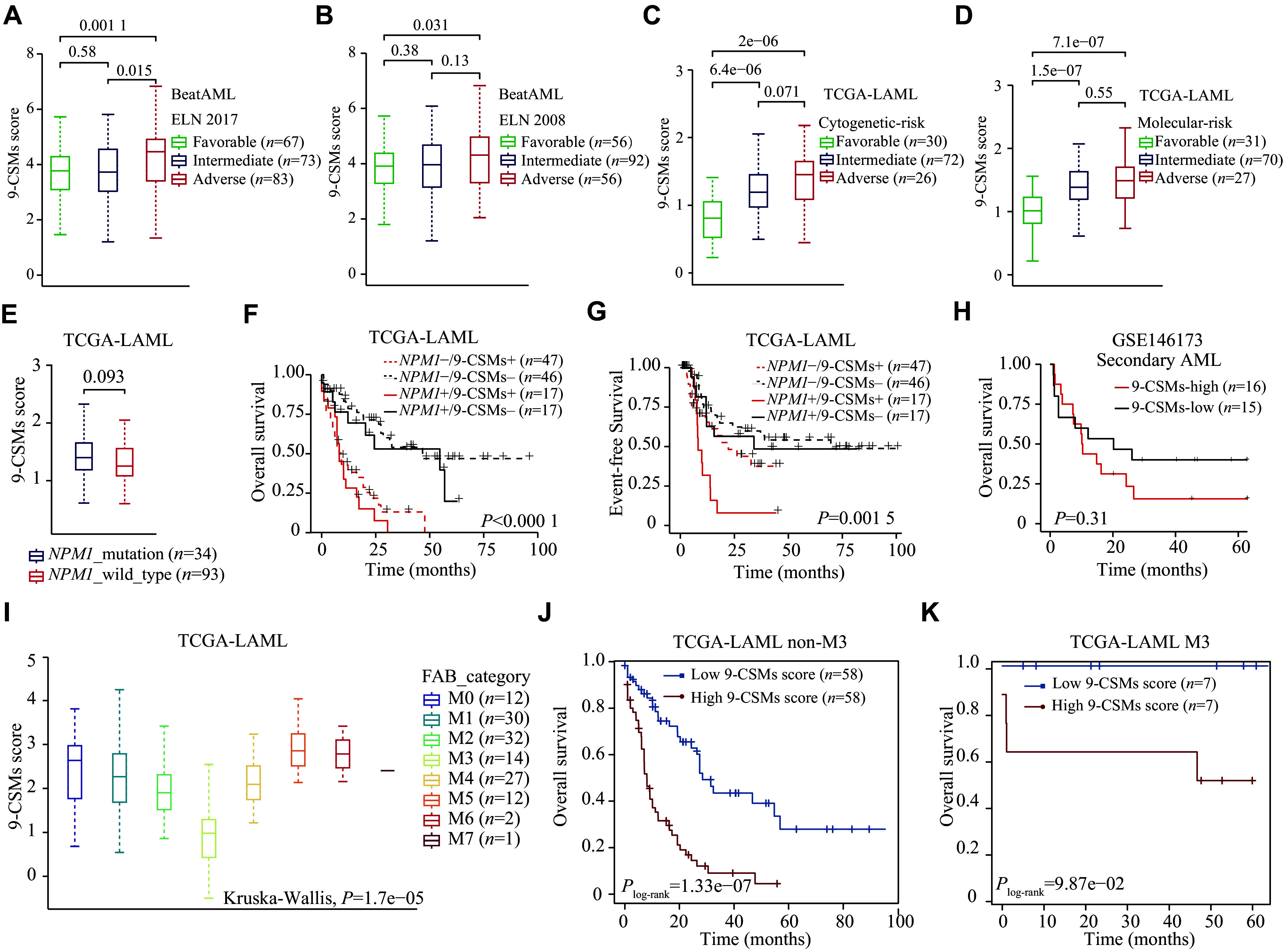
Evaluation of the 9-CSMs prognostic model in different AML risk stratifications and subtypes.

In both the BeatAML (239 patients) and TCGA-LAML (130 patients) cohorts, patients in the adverse risk group had a higher 9-CSMs risk score than those in the favorable and intermediate-risk groups ( ***Fig. 5A***– ***5D***). Furthermore, we found that the 9-CSMs risk score did not significantly differ between 34 *NPM1* mutated and 93 wild-type AML patients ( ***Fig. 5E***), and that the 9-CSMs risk score was shown to effectively predict prognosis in AML patients, regardless of their *NPM1* mutation status ( ***Fig. 5F*** and ***5G***). Additionally, we compared the 9-CSMs risk score between *de novo* and secondary AML patients using the GSE183817 (4 *de novo* and 3 secondary AML patients) and GSE146173 (202 *de novo* and 31 secondary AML patients) datasets and found that there was no significant difference in the 9-CSMs risk score between patients classified as *de novo* or secondary AML ( ***Supplementary Fig. 3A*** and ***3B***, available online). A higher 9-CSMs score in patients with secondary AML had a relatively poor prognosis but was not statistically significant, possibly because only 31 secondary AML samples were present in the GSE146173 ( ***Fig. 5H***). These findings indicate that the 9-CSMs prognostic model may be applicable to both *de novo* and secondary AML patients.

Acute promyelocytic leukemia is classified as the M3 subtype by the French-American-British classification system ^[ 25] ^. This subtype is sensitive to differentiation induction therapy containing *all-trans* retinoic acid and arsenic trioxide ^[ 26] ^. In the current study, we found that 14 AML patients with M3-type had the lowest 9-CSMs risk score ( ***Fig. 5I***). To exclude the influence of patients with M3 type, we performed survival analysis in 116 non-M3 AML patients and found that the 9-CSMs risk score might still be used for prognostic stratification of these patients ( ***Fig. 5J***). Notably, the 9-CSMs risk score also provided prognostic stratification in M3 patients ( ***Fig. 5K***). These results indicate that the prognostic role of 9-CSMs risk score may be independent of AML subtypes.

### Patients with high 9-CSMs risk scores were resistant to chemotherapy

**Figure 6:**
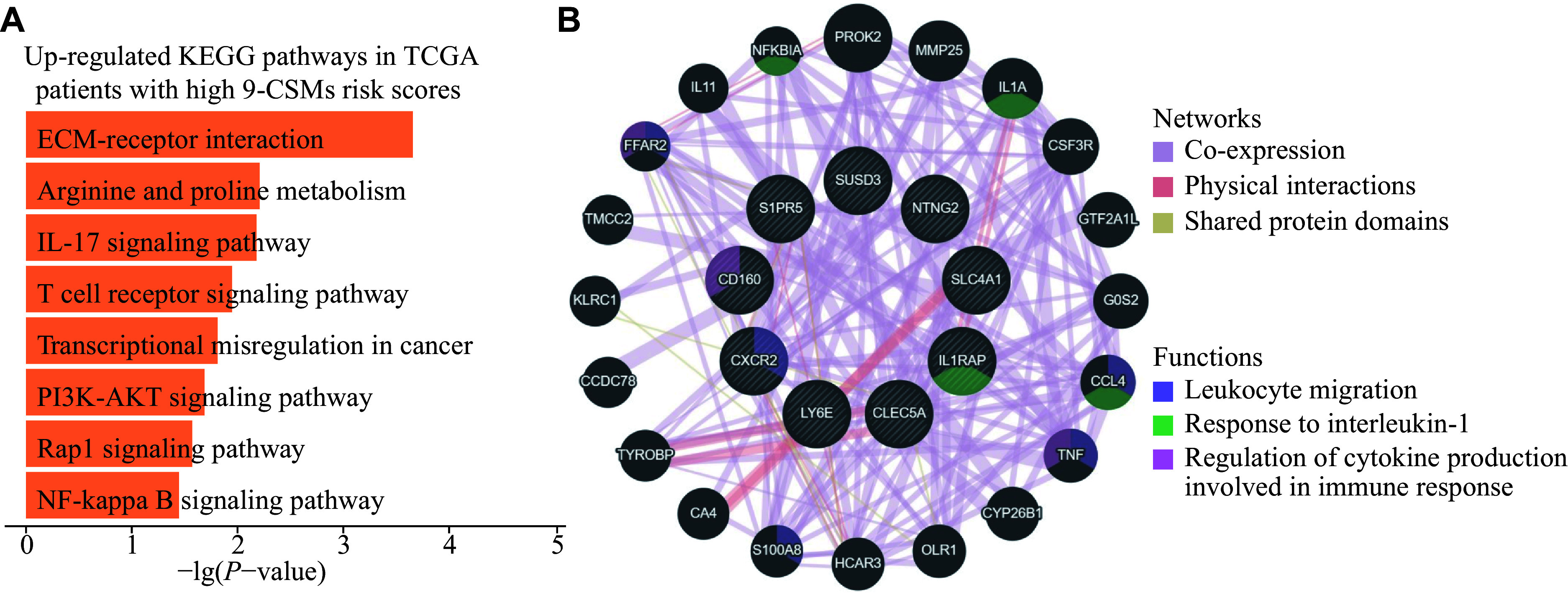
Functional enrichment and gene co-expression network analysis of the 9-CSMs prognostic model.

To further characterize patients with high 9-CSMs risk scores, we classified the patients in the TCGA-LAML training cohort according to the median 9-CSMs risk score and performed a functional enrichment analysis. We observed eight up-regulated KEGG pathways in patients with high 9-CSMs risk scores ( ***Fig. 6A***). Notably, one of these enriched signaling pathways, the PI3K-AKT signaling pathway, has been reported to be frequently activated in AML patient blasts and contributes to drug resistance of these cells ^[ 27] ^. Additionally, we performed a network analysis based on physical interactions and co-expression and identified that 9-CSMs gene modules were associated with leukocyte migration ( ***Fig. 6B***). It is noteworthy that leukocyte migration was reported to be associated with extramedullary metastasis in AML ^[ 28] ^, and one study has proposed a correlation between extramedullary infiltration in AML and a poorer prognosis ^[ 29] ^.

Based on the prognostic value of 9-CSMs, we hypothesized that the 9-CSMs prognostic model might help predict the treatment outcome of patients. To test this hypothesis, we investigated the association between 9-CSMs risk scores and drug resistance using real-world medication data of AML patients.

**Figure 7:**
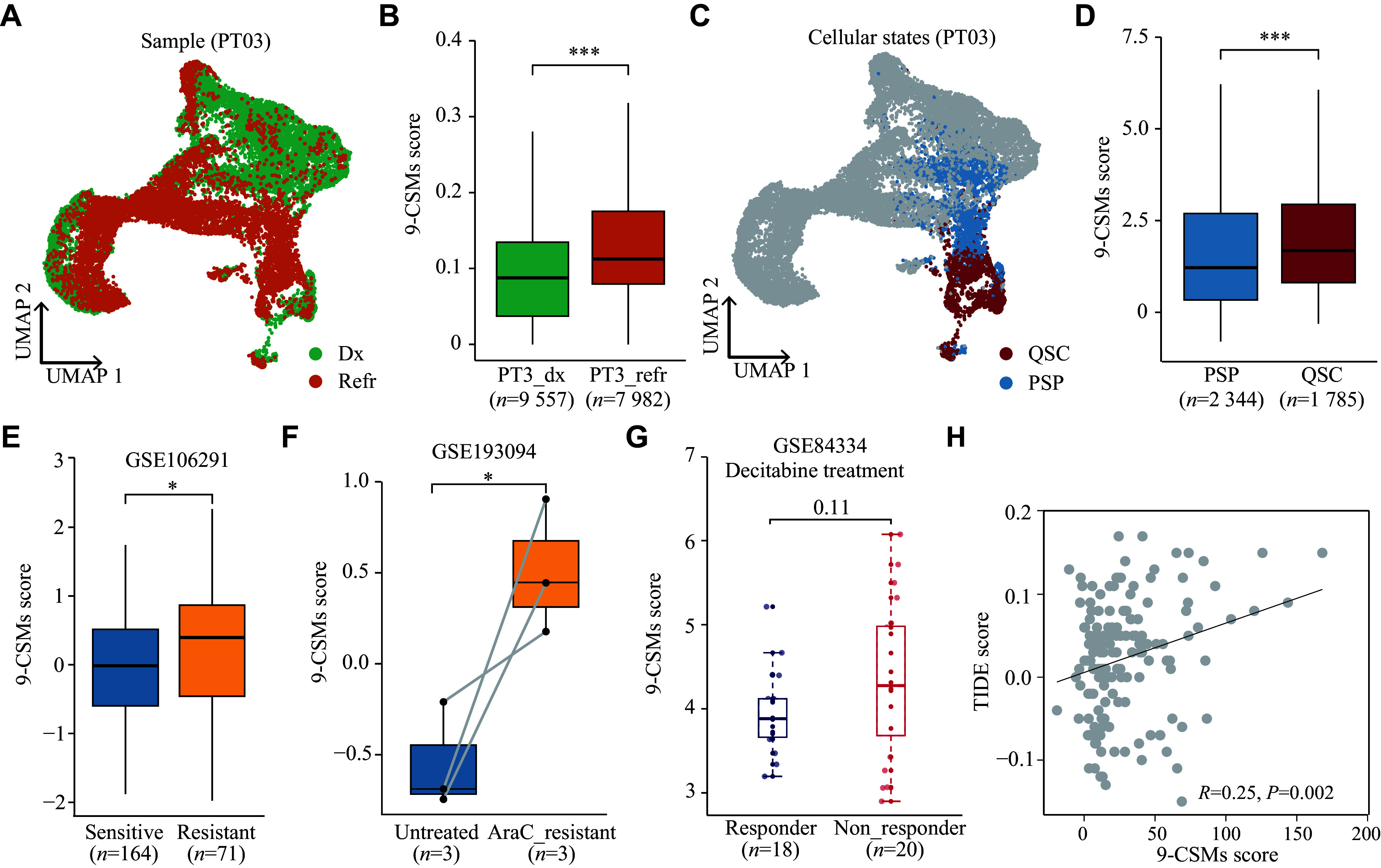
Relationship between 9-CSMs risk score and drug resistance in real-world medication data of AML patients.

Firstly, we derived malignant cells from published AML scRNA-seq datasets and analyzed the association between 9-CSMs risk scores in these cells and their sensitivity to chemotherapy, the prevailing treatment for AML ^[ 22] ^. The results showed that malignant cells that refractory to chemotherapy ( *n* = 7982) had higher 9-CSMs risk scores than those at the initial diagnosis ( *n* = 9557) ( ***Fig. 7A*** and ***7B***). We also found that quiescent stem-like cells (QSCs, *n* = 1785) had a higher 9-CSMs risk score than proliferating stem/progenitor-like cells (PSPs, *n* = 2344) ( ***Fig. 7C*** and ***7D***), which was consistent with a previous research showing that a subpopulation of QSCs was involved in chemoresistance and poor outcome in AML compared with PSPs ^[ 22] ^.

Furthermore, we found that the 9-CSMs score was higher in the chemotherapy-resistant group ( *n* = 71) than in the chemotherapy-sensitive group in the GSE106291 dataset ( *n* = 164) ( ***Fig. 7E***). Additionally, the 9-CSMs score was higher in the cytarabine-resistant group ( *n* = 3) than in the group without treatment ( *n* = 3) in the GSE193094 dataset ( ***Fig. 7F***). We subsequently evaluated the response of patients with high 9-CSMs scores to the hypomethylating agent, decitabine. Patients who did not respond to decitabine ( *n* = 20) had higher 9-CSMs risk scores than those who responded to decitabine ( *n* = 18) in the GSE84334 dataset, but this difference was not statistically significant ( ***Fig. 7G***). We also predicted the response of AML patients to the existing ICIs targeting PD-1 and CTLA-4 using the online TIDE tool, which integrates both intrinsic tumor cell characteristics and the tumor microenvironment to assess the response to immunotherapy. The result showed a significant positive correlation between the 9-CSMs risk score and the TIDE score ( ***Fig. 7H***), indicating that patients with a high 9-CSMs risk score tended to be resistant to ICIs.

In summary, patients with high 9-CSMs scores may respond poorly to conventional chemotherapy, hypomethylating agents, and the existing ICI treatment. These results indicate that the 9-CSMs prognostic model may be a valuable tool for predicting patient response to the treatments.

### Potential drug prediction in patients with higher 9-CSMs scores

**Figure 8:**
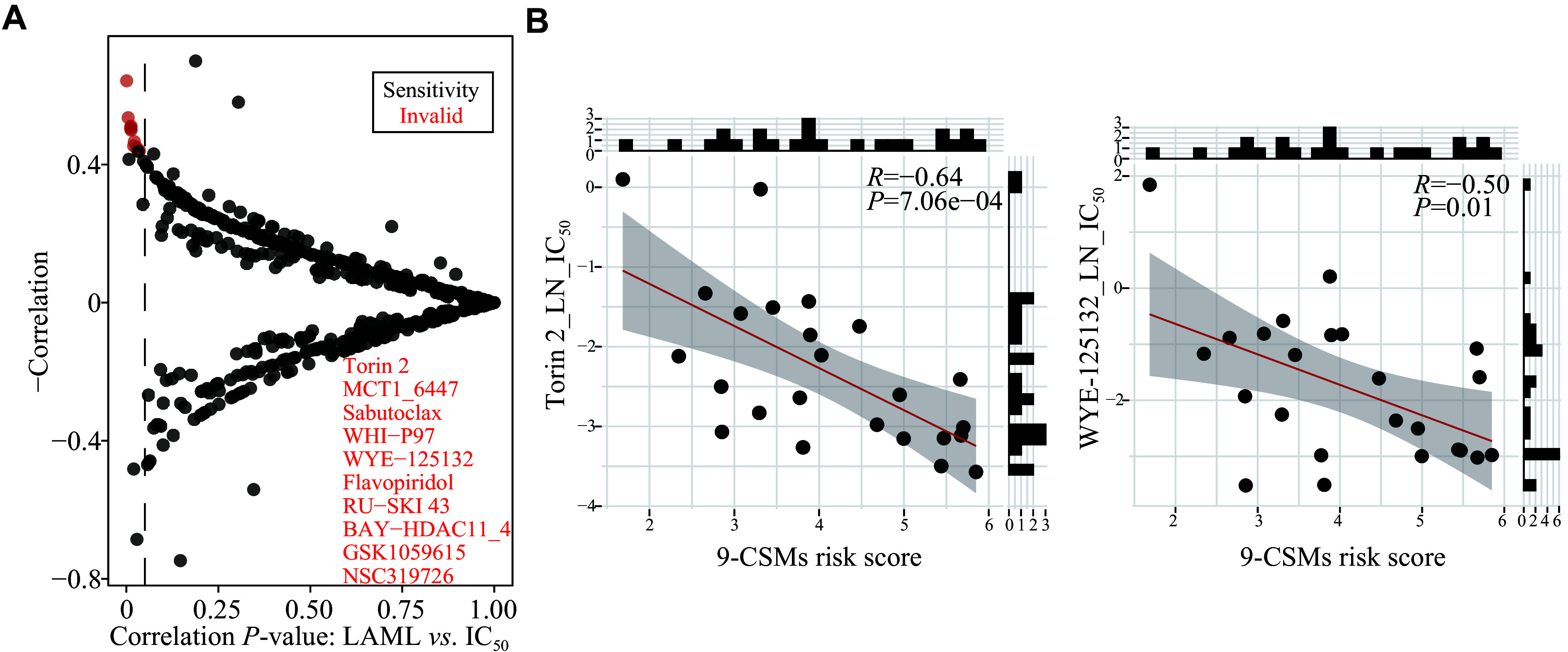
Prediction of potential drugs in patients with higher 9-CSMs scores.

To further determine the clinical relevance of the 9-CSMs risk score in terms of AML patient treatment, we analyzed the association between the CSM prognostic score and drug sensitivity to small-molecule drugs using the drug IC _50_ data and cell line expression data from the GDSC database. The results showed that patients with a high prognostic risk score were more sensitive to Torin 2, WYE-125132, and GSK1059615 ( ***Fig. 8A*** and ***8B***, ***Supplementary Fig. 4A ***– ***4C*** [available online]). Torin 2 and WYE-125132 act as inhibitors of mTOR ^[ 30– 31] ^, a downstream target of the PI3K/AKT pathway, while GSK1059615 is a dual inhibitor of PI3K and mTOR ^[ 32] ^. In gastric cancer, GSK1059615 was reported to block the PI3K-AKT-mTOR cascade activation; and the administration of GSK1059615 in nude mice significantly suppressed the growth of subcutaneous AGS xenografts ^[ 32] ^. Combined with the aforementioned results that TCGA-AML patients with high 9-CSMs risk scores exhibited a high activation of the PI3K-AKT signaling pathway, we believe that the 9-CSMs prognostic model may play a role in guiding the selection of chemotherapy drugs for patients.

## Discussion

The diagnostic and screening advantages of CSFs have been found in various cancers, including AML. However, CSFs for predicting the prognosis of AML patients still need to be explored. Here, we constructed a prognostic model based on nine CSMs ( *SLC4A1*, *CLEC5A*, *IL1RAP*, *CD160*, *S1PR5*, *LY6E*, *NTNG2*, *CXCR2*, and *SUSD3*) that might provide a reliable prognosis value for clinical application. Our results indicate that the risk score may stratify AML patients into two risk groups exhibiting significantly divergent survival outcomes.

Firstly, we examined the difference in the expression of CSMs between AML patients and healthy controls, which was associated with overall survival in AML patients. The functional enrichment analysis showed that the differentially up-regulated CSMs were correlated with the inflammatory response, IL-6/JAK/STAT3 signaling pathway, and IL-2/STAT5 signaling pathway. These pathways were reported to be dysregulated in many cancers, indicating that CSMs play a crucial role in cellular immunity. Secondly, we constructed a prognostic model based on multi-model analysis, and this 9-CSMs prognostic score was found to be an independent prognostic factor for AML patients. We also evaluated the performance of the prognostic model and validated its accuracy at both the transcriptome and proteome levels. Thirdly, paired single-cell data showed that refractory samples in malignant cells had higher risk scores than those at the initial diagnosis. Finally, we examined the correlation between the risk score of our prognostic model and drug sensitivity. We found that AML patients in a high-risk category were more likely to respond to treatments, such as Torin 2, WYE-125132, and GSK1059615, demonstrating the clinical importance of the 9-CSMs prognostic model in the treatment selection of AML patients in the future. Collectively, we constructed an effective prognostic model that might significantly contribute to prognosis prediction and serve as a crucial reference in clinical treatment.

The use of immunotherapies, especially ICIs, has demonstrated effects in enhancing anti-tumor responses in certain solid tumors and hematological cancers ^[ 33] ^. However, several studies have reported that the existing ICIs, such as PD-1 and CTLA-4 inhibitors, have limited efficacy in the treatment of AML patients. Moreover, it was reported that AML patients who received hematopoietic stem cell transplantation developed severe graft-versus-host disease after ICI treatment ^[ 34– 35] ^. Thus, it is urgent to identify new immunotherapies for AML patients. To address this need, our model included five risk factors: CXCR2, LY6E, SUSD3, S1PR5, and IL1RAP. High expression of *IL1RAP* is independently associated with low overall survival in patients with AML, and IL1RAP may serve as a potential immunotherapeutic target for malignancies ^[ 36] ^. Meanwhile, CXCR2, produced by tumors, induces neutrophil extracellular traps that interfere with immune cytotoxicity ^[ 37] ^; thus, inhibiting CXCR2 may significantly reduce neutrophil infiltration and enhance anti-tumor T cell activity *via* promoting CD8 T cell activation ^[ 38] ^. Furthermore, the increased expression of *LY6E* was also correlated with the increased expression of immune checkpoint molecules, PD-L1 and CTLA-4, as well as the reduced activation of natural killer cells ^[ 39] ^. These findings suggest that CXCR2, LY6E, and IL1RAP may provide new targets for immunotherapy in AML patients.

Conversely, our model also included four protective factors: *CD160*, *CLEC5A*, *NTNG2*, and *SLC4A1*. CD160, which is highly expressed in natural killer cells, plays a significant role in killing tumor cells. Studies have shown that CD160 factors regulate functions of natural killer cells and control potential early cancer therapy ^[ 40] ^. CLEC5A is a cell surface receptor associated with the activation and differentiation of myeloid cells. In the current study, we report for the first time that *NTNG2* is highly expressed in AML. Although NTNG2 is not well reported in AML, it is associated with the development of other cancers, such as breast and thyroid cancer ^[ 41] ^.

**Figure 9:**
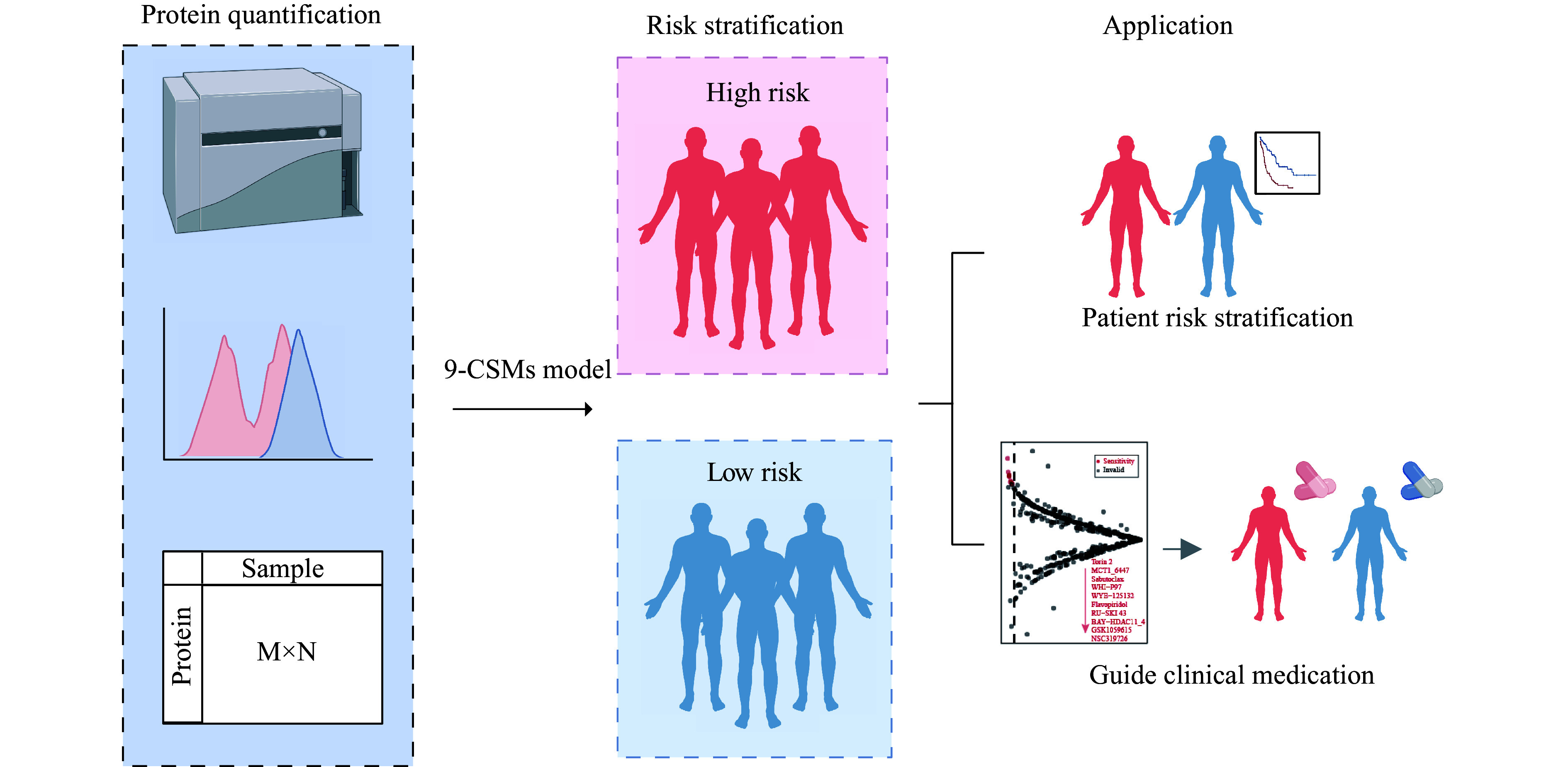
Clinical applications of the 9-CSMs risk score.

In the current study, we used high-throughput transcriptomic data to comprehensively assess the effects of all CSMs on the prognosis of 130 AML patients from TCGA-LAML, and ultimately constructed a 9-CSMs prognostic model associated with AML patient outcomes. In clinical practice, the expression levels of each CSM may be obtained by some protein quantitative methods, such as cytometry by time-of-flight (CyTOF). Then, protein quantification data may be used to calculate the 9-CSMs risk score using the coefficients reflecting the correlation of each marker with disease prognosis derived from extensive clinical research and statistical analysis in the current study. The 9-CSMs score may help us stratify patients by risk, provide personalized treatment plans, aid in the development of targeted new drugs, and accelerate the process of bringing new therapies to market ( ***Fig. 9***).

However, the current study has several limitations. 1) We did not specifically identify the direct molecular relationships between CSMs and the progress in AML patients. The deep mechanisms of how CSMs reflect prognosis, such as the cell-cell interaction, biological processes in cells, and cellular signal transduction, remain unclear. 2) In addition, we trained and validated the prognostic model entirely based on public databases. We would focus on conducting a prospective study to validate our findings experimentally in the future. 3) Moreover, the risk definition of 9-CSMs risk score in individual patients is unclear, and we need to collect more quantitative protein data to evaluate the association between 9-CSMs risk score value and prognosis to determine the appropriate threshold for clinical application.

In summary, we identify a 9-CSMs prognostic model that may compute the risk score and predict AML prognosis accurately. The predictive value of the 9-CSMs prognostic model is validated at both transcriptomic and proteomic levels, establishing it as an independent prognostic factor for AML patients. Further analysis indicates that patients with high 9-CSMs risk scores are resistant to chemotherapy and that inhibitors of PI3K are potential therapeutic drugs for patients with high 9-CSMs scores. Overall, our research will provide valuable information for predicting prognosis and guiding therapeutic decisions for AML patients.

## SUPPLEMENTARY DATA

Supplementary data to this article can be found online.
