# Supplementary material for: Identification of cell surface markers for acute myeloid leukemia prognosis based on multi-model analysis
Source: J Biomed Res. 2024 May 29;38(4):397–412. doi: 10.7555/JBR.38.20240065 (PMC11300515; doi:10.7555/JBR.38.20240065)
Supplement: Supplementary file 1 — Supplementary data to this article can be found online. [file jbr-38-4-397-S1.pdf]

Available online at www.jbr-pub.org.cn

Open Access at PubMed Central

Journal of Biomedical Research, 2024 0(000): 1–3

Supplementary Data

JBR

Identification of cell surface markers for acute myeloid leukemia prognosis based on multi-model analysis

Jiaqi Tang[1,2,3,△], Lin Luo[1,2,3,△], Bakwatanisa Bosco[1,3,△], Ning Li[1,3], Bin Huang[1,3], Rongrong Wu[1,3], Zihan Lin[1,3], Ming Hong[4,5], Wenjie Liu[4,5], Lingxiang Wu[1,3], Wei Wu[1,3], Mengyan Zhu[1,3], Quanzhong Liu[1,3], Peng Xia[1,3], Miao Yu[1,3], Diru Yao[1,3], Sali Lv[1,3], Ruohan Zhang[1,3], Wentao Liu[6,✉], Qianghu Wang[1,3,7,✉], Kening Li[1,2,✉]

[1]*Department of Bioinformatics, School of Biomedical Engineering and Informatics, Nanjing Medical University, Nanjing, Jiangsu 211166, China;*
[2]*Department of Hematology of the Affiliated Huai'an No. 1 People's Hospital of Nanjing Medical University, Northern Jiangsu Institute of Clinical Medicine, Huai'an, Jiangsu 223300, China;*
[3]*Collaborative Innovation Center for Personalized Cancer Medicine, Jiangsu Key Lab of Cancer Biomarkers, Prevention and Treatment, Nanjing Medical University, Nanjing, Jiangsu 211166, China;*
[4]*Department of Hematology, the First Affiliated Hospital of Nanjing Medical University, Jiangsu Province Hospital, Nanjing, Jiangsu 210029, China;*
[5]*Key Laboratory of Hematology of Nanjing Medical University, Nanjing, Jiangsu 210029, China;*
[6]*Department of Pharmacology, School of Basic Medical Sciences, Nanjing Medical University, Nanjing, Jiangsu 211166, China;*
[7]*The Affiliated Cancer Hospital of Nanjing Medical University, Jiangsu Cancer Hospital, Jiangsu Institute of Cancer Research, Nanjing, Jiangsu 210002, China.*

Supplementary Tables

Supplementary Table 1 (available online) shows the patient characteristics for the TCGA training data and the GEO external validation datasets.

Supplementary Table 2 (available online) shows nine CSMs determined by multivariate Cox regression.

Supplementary Table 3 (available online) shows the list of 2 886 surfaceome proteins.

Supplementary Table 4 (available online) shows the genes differentially expressed between AML andnormal samples.

Supplementary Table 5 (available online) shows the significantly differentially expressed genes retained by univariate Cox regression analysis of TCGA data.

Supplementary Table 6 (available online) shows the CSMs selected by LASSO regression.

Supplementary Table 7 (available online) shows the CSMs selected by adaptive LASSO regression.

Supplementary Table 8 (available online) shows the CSMs selected by Elastic Net regression.

Supplementary Table 9 (available online) shows CSMs identified by multi-model selection analysisbased on the smallest AIC.

Supplementary Table 10 (available online) shows the description of 9-CSMs prognostic model.

Supplementary Table 11 (available online) shows 9-CSMs risk scores of TCGA-LAML.

Supplementary Table 12 (available online) shows 9-CSMs risk scores of GSE10358.

Supplementary Table 13 (available online) shows

△These authors contributed equally to this work.

✉Corresponding authors: Wentao Liu, Department of Pharmacology, School of Basic Medical Sciences, Nanjing Medical University, 101 Longmian Avenue, Nanjing, Jaingsu 211166, China. E-mail: painresearch@njmu.edu.cn; Qianghu Wang and Kening Li, Department of Bioinformatics, School of Biomedical Engineering and Informatics, Nanjing Medical University, 101 Longmian Avenue, Nanjing, Jiangsu, 211166, China. E-mails: wangqh@njmu.edu.cn (Wang) and likening@njmu.edu.cn (Li).

Received: 18 March 2024; Revised: 21 May 2024; Accepted: 23 May 2024; Published online: 29 May 2024

CLC number: R733.71, Document code: A

The authors reported no conflict of interests.

This is an open access article under the Creative Commons Attribution (CC BY 4.0) license, which permits others to distribute, remix, adapt and build upon this work, for commercial use, provided the original work is properly cited.

© 2024 by Journal of Biomedical Research.

https://doi.org/10.7555/JBR.38.20240065

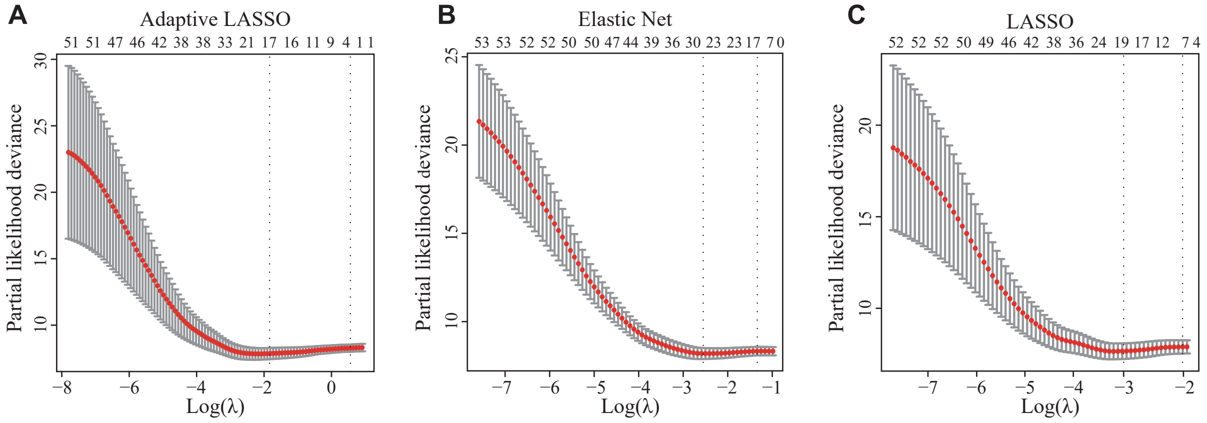

Supplementary Fig. 1 **Identification of prognostic 9-CSMs and construction of a prognostic model in the TCGA-LAML training cohort.** A plot of the parameters of the penalty term is used to select the value of λ, with the log(λ) value on the abscission and the degrees of freedom on the ordinate. Cross-validation for tuning the feature selection using the Adaptive LASSO (A), Elastic Net(B), and LASSO regression analyses. The base of log(λ) is Euler's number (e).

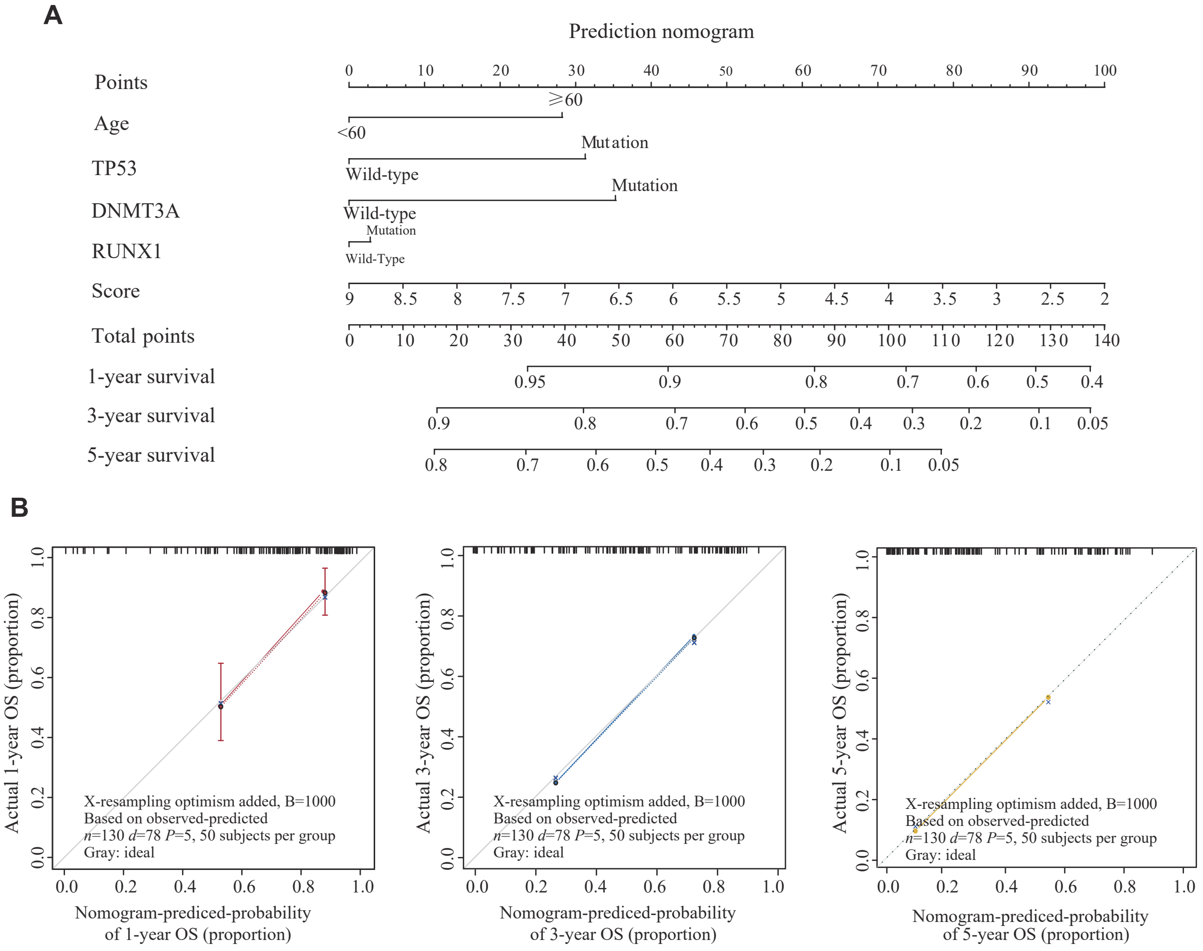

Supplementary Fig. 2 **Construction and calibration of the prediction nomogram.** A: The nomogram model incorporates the independent prognostic variables including the 9-CSMs risk score, age, *TP53, RUNX1*, and *DNMT3A, P* < 0.05. B: Calibration curves for 1-year, 3-year, and 5-year overall survival of the predictive nomogram.

9-CSMs risk scores of GSE12417.

Supplementary Table 14 (available online) shows 9-CSMs risk scores of GSE71014.

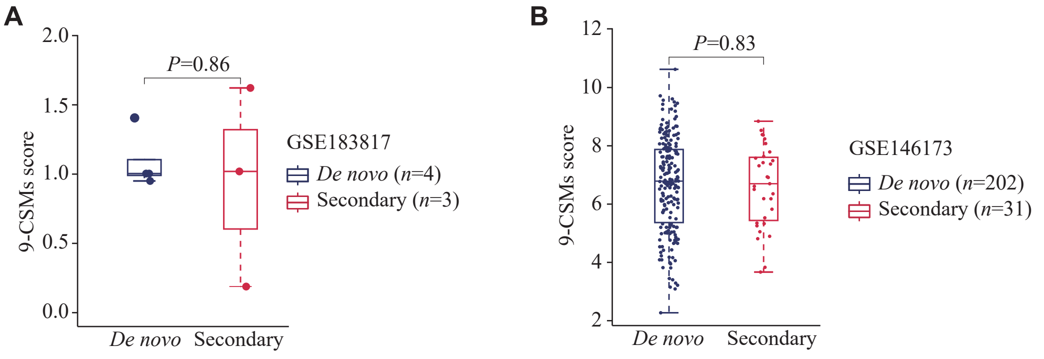

Supplementary Fig. 3 **The relationship between 9-CSMs risk score and *de novo*/secondary AML patients.** A: Boxplot showing the 9-CSMs risk score among *de novo* and secondary AML patients in the BeatAML dataset. B: Boxplot showing the 9-CSMs risk scores among *de novo* and secondary AML patients in the GSE146173 dataset. Data are presented as the median and interquartile range. Wilcoxon rank-sum test was used to measure the differences between groups.

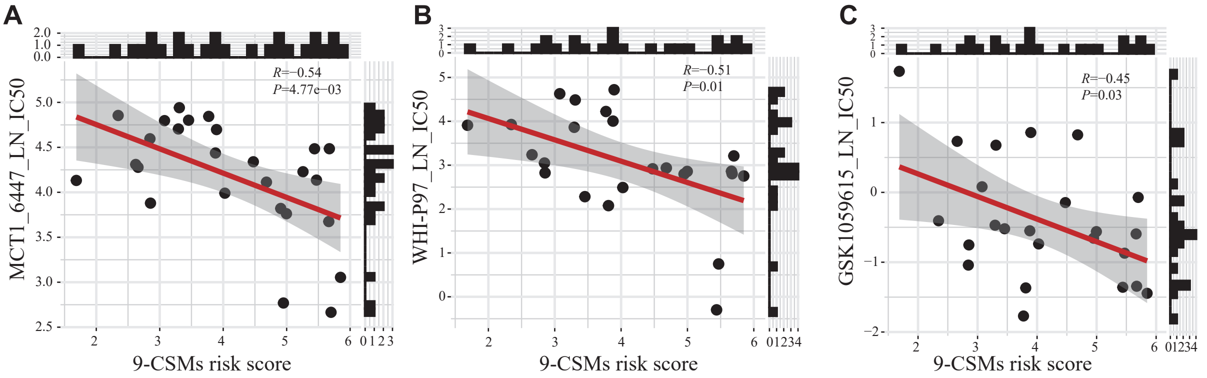

Supplementary Fig. 4 **The correlation analysis between 9-CSMs risk score and drug sensitivity.** A–C: The diagram of correlation shows the correlation between Genomics of Drug Sensitivity in Cancer (GDSC) drug sensitivity values (IC_{50}) and the 9-CSMs risk score for the most sensitive drugs. Abbreviation: CSMs, cell surface markers.
